# Quality of Life and Post-Operative Pain Following Laparoscopic Inguinal Hernia Repair With Self-Fixating Mesh: a Prospective Observational Study

**DOI:** 10.3389/jaws.2025.15203

**Published:** 2025-08-29

**Authors:** Samuel Josias Bizerra Calderon, Humberto Fenner Lyra, Tiago Rafael Onzi, Gilberto Kremer, Fernando Ferraz de Miranda, Getúlio Rodrigues de Oliveira Filho

**Affiliations:** ^1^ Ultralitho Medical Centre, Florianópolis, Brazil; ^2^ University Hospital, Federal University of Santa Catarina, Florianópolis, Brazil; ^3^ Department of Surgery, Federal University of Santa Catarina, Florianópolis, Brazil

**Keywords:** hernia, inguinal, laparoscopy, surgical mesh, quality of life, post-operative pain

## Abstract

**Background:**

Laparoscopic inguinal hernia repair using self-fixating mesh has been associated with advantages such as reduced post-operative pain and fewer complications. However, chronic pain and quality-of-life outcomes remain concerns. Objective: To evaluate post-operative quality of life and pain following transabdominal preperitoneal (TAPP) hernia repair using self-fixating mesh.

**Methods:**

This prospective observational study included 90 patients undergoing TAPP hernia repair in a institution in Brazil between 2023 and 2025. Quality of life was assessed using the EuraHS-QoL questionnaire at baseline, 1, 3, and 6 months post-operatively. Pain was measured using a numeric rating scale. Descriptive and inferential statistical analyses, including linear mixed models, were applied.

**Results:**

Most patients were male (94.4%) with a mean age of 57.9 years. EuraHS-QoL scores improved significantly at 3 and 6 months post-operatively compared to baseline (p < 0.001). No significant improvement was noted at 1 month. Pain and cosmetic domain scores improved early, while the restriction domain showed delayed improvement. Chronic pain rates at 3 months were among the lowest reported in the literature.

**Conclusion:**

TAPP hernia repair with self-fixating mesh resulted in significant quality-of-life improvement beginning at 3 months post-operatively. The self-fixating mesh technique demonstrated favorable outcomes, including low chronic pain incidence.

## Introduction

A hernia is the protrusion of a viscus from the cavity that normally contains it, through an orifice, an anatomical canal, or any other gap, being a very common condition that affects all age groups, with a lifetime risk of 27% for men and 3% for women [1]. Inguinal hernia repair is one of the most commonly performed surgeries by general surgeons [2]. Over 20 million patients are operated annually worldwide for inguinal hernia [3]. In Brazil’s public health system alone, 182,570 inguinal hernia surgeries were performed in 2023 [4].

The treatment of symptomatic inguinal hernia is surgical. Among the various available techniques, the open approach popularized by Lichtenstein and laparoscopic techniques—particularly the totally extraperitoneal (TEP) and the transabdominal preperitoneal (TAPP)—are the most widely used [5].

Currently, laparoscopic inguinal surgery has gained considerable space in the treatment of inguinal hernia, with advantages such as reduced acute post-operative pain, lower rates of chronic post-operative pain, fewer surgical site infections, faster recovery, and comparable or even lower recurrence rates when compared to the open approach [6–8].

Between the laparoscopic techniques, TEP and TAPP offer similar results regarding surgical time, length of hospital stay, early return to activities, reduced post-operative pain, and recurrence rate. However, each technique has specific risks: visceral injury in TAPP and vascular injury in TEP [9, 10].

The use of mesh prosthesis is standard in the treatment of inguinal hernia, having originated from Lichtenstein’s intuition and the theory of tension-free surgery, significantly reducing recurrence rates [11]. Over time, meshes have evolved in terms of materials and weight, reaching a certain standard with highly acceptable outcomes [12]. However, stabilization of the mesh with some type of fixation is recommended. Among the laparoscopic options are permanent metallic staples, absorbable tacks, fibrin glue, and self-fixating mesh [13, 14].

The ProGrip Laparoscopic™ Self-fixating Mesh (Medtronic™, Trévoux, France) is a self-adhering prosthetic material made of polyethylene terephthalate with absorbable polylactic acid microgrips. It is a macroporous mesh with an initial weight of 82 g/m^2^ that decreases to 49 g/m^2^ after absorption of the microgrips [15].

There is a growing tendency in the literature to avoid traumatic fixation methods, such as tacks, in favor of atraumatic methods like glue or self-fixating meshes to prevent acute and especially chronic pain caused by inadvertent nerve entrapment during mesh fixation [16–18].

Among post-operative complications, chronic post-operative pain has the greatest impact on quality of life. Chronic pain is defined as moderate to severe pain that persists beyond 3 months post-surgery. Recently, the HerniaSurge Group recommended that moderate pain affecting daily activities for more than 3 months also be classified as chronic [19, 20].

The incidence of moderate to severe chronic pain after mesh repair is reported in 10%–12% of patients, attributed to multiple factors, including nerve injury or entrapment during mesh fixation [13, 21].

Laparoscopic repair tends to present lower rates of chronic post-operative pain compared to open repair. However, chronic pain remains a challenge in laparoscopic techniques as well, with reported rates ranging from 3.3% to 20% [15, 22, 23].

When evaluating laparoscopic hernia repair, non-traumatic fixation methods such as glue or self-fixating mesh have shown advantages in reducing chronic pain compared to tack fixation [18, 19, 24, 25].

Currently, quality-of-life evaluation is a growing concern across various health disciplines [26]. The 36-Item Short Form Survey (SF-36) is widely used for assessing general quality of life [27].

More recently, disease-specific questionnaires have been developed, such as the Carolinas Comfort Scale, a validated hernia-specific instrument that evaluates pain, mesh sensation, and movement limitations on a 0–5 scale [28].

In 2016, the European Hernia Society developed and published the EuraHS-QoL questionnaire, a patient-reported outcome measure specifically designed for hernia surgery. It consists of only nine questions across three domains: pain, activity restriction, and cosmetic discomfort. The tool has been validated in Portuguese as well [16, 26, 29].

This study aimed to assess post-operative quality-of-life and pain progression in patients undergoing laparoscopic transabdominal preperitoneal (TAPP) inguinal hernia repair using self-fixating mesh (Medtronic™).

The primary objective was to evaluate changes in quality of life using the EuraHS-QoL score in patients undergoing TAPP hernia repair with self-fixating mesh. The secondary objective was to assess post-operative pain intensity at three and 6 months.

## Materials and Methods

### Study Design

This was a prospective observational study conducted at the Department of Surgery of the Federal University of Santa Catarina and at Ultralitho Medical Centre in Florianópolis, SC, between November 2023 and February 2025. The study was approved by the Ethics Committee of the Federal University of Santa Catarina under the registration CAAE: 73696523.0.0000.0121. Informed consent was obtained from all participants.

### Sample

A total of 90 patients with primary inguinal hernia who underwent laparoscopic transabdominal preperitoneal (TAPP) repair using a self-fixating mesh (Progrip Lap™; Medtronic, [Trévoux, France]) were included.

Inclusion criteria were patients of both sexes, over 18 years of age, diagnosed with unilateral or bilateral inguinal or femoral hernia, and undergoing laparoscopic TAPP repair using only the self-fixating mesh (Progrip Lap™; Medtronic, C Trévoux, France). Exclusion criteria included: patients under 18 years of age; patients with recurrent hernia after laparoscopic repair; large inguinal hernias with defect size >4 cm; surgeries using any form of additional mesh fixation; incarcerated hernias or emergency surgeries; and patients classified as ASA IV (American Society of Anesthesiologists).

### EuraHS-QoL Quality of Life Score

The EuraHS-QoL is a validated quality of life score used specifically in patients diagnosed with inguinal hernia. It comprises three domains: pain, restriction, and cosmetic appearance. The pain domain includes three items scored from 0 (no pain) to 10 (worst possible pain), for a total of 30 points. The restriction domain includes four items, each scored from 0 (no restriction) to 10 (total restriction), with a total of 40 points. The cosmetic domain comprises two items scored from 0 (best appearance) to 10 (worst appearance), totaling 20 points. The overall score ranges from 0 to 90, with higher values indicating worse quality of life. This questionnaire was applied pre-operatively and at 1, 3, and 6 months post-operatively [16].

### Post-Operative Pain

Post-operative pain was assessed during the first week and at 3 and 6 months post-operatively using an 11-point numeric rating scale (NRS), where 0 corresponds to no pain and 10 to the worst imaginable pain. Persistent moderate pain at 3 months post-operatively was defined as an NRS value greater than 3 [30].

### Variables

Pre-operative variables included: age, gender, body mass index (BMI), ASA classification (American Society of Anesthesiologists), smoking status, comorbidities (systemic arterial hypertension, diabetes mellitus, chronic obstructive pulmonary disease, liver disease, kidney disease, aortic aneurysm), history of non-inguinal chronic pain (such as fibromyalgia, migraine, joint pain, post-traumatic pain, post-operative pain, oncologic pain, and nerve compression/injury pain), as well as hernia location and associated symptoms.

Intraoperative variables included: hernia classification according to the European Hernia Society system, mesh size (15 × 10 cm or 16 × 12 cm), surgical time, and surgical complications.

Post-operative variables included: pain during the first post-operative week (assessed using the numeric pain rating scale), immediate complications such as haematoma, acute bleeding, intense pain, and haematuria, and complications within 1 month after surgery (seroma, haematoma, pain, and recurrence).

### Data Collection and Storage

Data collection and administration of the quality-of-life questionnaire were conducted in person during the pre-operative consultation, in the operating room, during hospital stay, and during post-operative follow-up by a nurse research assistant. Follow-up at one, three, and 6 months was conducted by telephone by the research assistant. Additionally, a face-to-face consultation was conducted on the 30th post-operative day.

Coded data were entered by the research assistant into stored in secure, anonymous platforms REDCap (Research Electronic Data Capture) and the European Hernia Society Registry (EHS Registry).

### Statistical Analysis

Descriptive analysis included frequency tables for categorical variables and mean, standard deviation, median, minimum and maximum values, and 25th and 75th percentiles for continuous variables.

The EuraHS-QoL quality of life questionnaire included a “not applicable” option in the restriction domain, used when a patient did not engage in a particular activity. Missing values due to “not applicable” responses were handled as follows: for the pain domain: if one item was missing, it was imputed using the average of the other two items; if two or three items were missing, the domain score was considered missing; for the restriction domain: if one or two items were missing, they were replaced with the average of the remaining items; if three or four items were missing, the domain score was considered missing; for the cosmetic domain: if one item was missing, it was replaced by the value of the other item; if both were missing, the domain score was considered missing. For the global score: if one domain score was missing, it was replaced by the average of the remaining two domain scores; if two domains were missing, the global score was considered missing [16].

A linear mixed model (LMM) was fitted to assess the evolution of quality of life scores (EuraHS-QoL) at different post-operative follow-up time points, and the same approach was used for numerical pain scale scores. Candidate predictor variables were evaluated individually using univariate linear mixed models, with time as a fixed effect and the patient as a random intercept. AIC values, marginal and conditional R^2^, and p-values were extracted to guide preliminary covariate selection. Selected variables were assessed for multicollinearity using the Pearson correlation matrix and variance inflation factor (VIF), with a VIF <5 considered acceptable. Variables showing high correlation (r > 0.6) were further evaluated and removed to avoid redundancy.

Subsequently, linear mixed models (LMM) were fitted using the *lmer()* function from the *lme4* package, modeling the EuraHS-QoL outcome variable over time. The random effects structure included patient-specific intercepts ((1 | patient_id)) to account for intra-individual dependency across repeated measures. Due to the skewed residuals in the original model, a Box-Cox transformation was applied to the outcome variable. The optimal lambda value was estimated via maximum likelihood and used for the transformation.

### Sample Size Calculation

The sample size was calculated for this descriptive observational study based on parameters established *a priori* to ensure adequate precision of the estimates. The calculation assumed a 95% range for the EuraHS-QoL score between 0 and 44 points on a 90-point scale. A margin of error for the mean of 2% (corresponding to 9.09% of the estimated mean) and a 99% confidence level were also adopted. The estimated mean EuraHS QoL score was 22 with a standard deviation of 11, as reported in the literature [16]. These parameters resulted in a minimum required sample size of 79 patients. To account for potential losses during follow-up, the study aimed to collect data from 90 patients.

The sample size calculation was performed using the online tool provided by The Donor Committee for Enterprise Development^1^ [31]. Data analysis was conducted using R software (version 4.4.3, R Foundation for Statistical Computing, Vienna, Austria).

## Results

### Patients and Procedures

**FIGURE 1:**
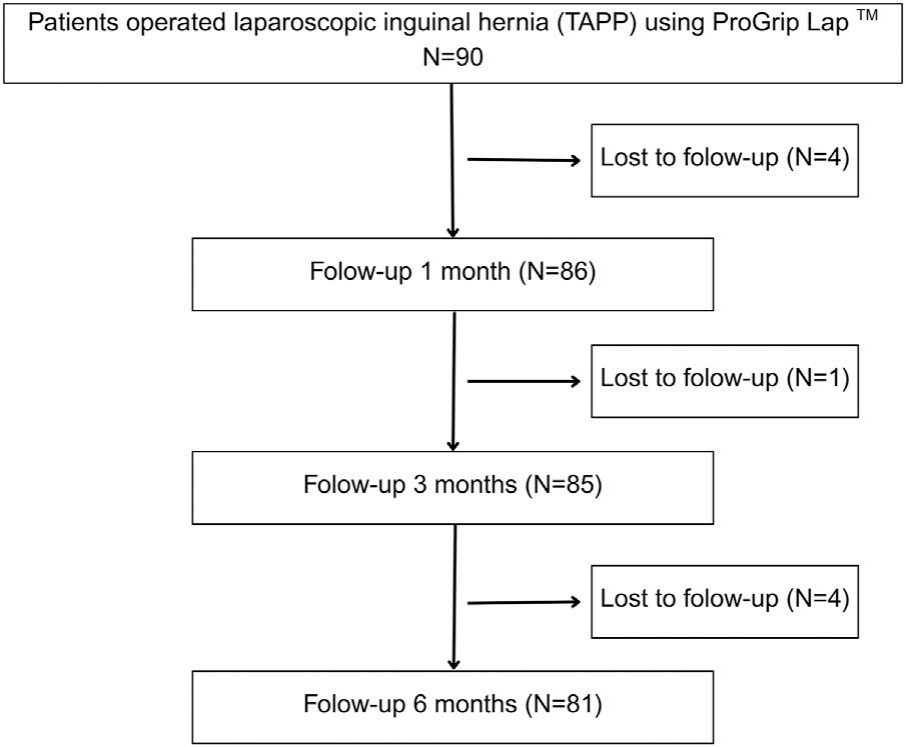
Study flow diagram (CONSORT).

A total of 90 patients were included in the study (Figure 1). Most were male (85, 94.4%), with a mean age of 57.9 years (standard deviation of 13.2), ranging from 31 to 83 years.

Demographic and clinical characteristics of the sample are shown in Table 1. In 64 patients (71%) the hernia defect was unilateral, and in 26 (28.9%) it was bilateral. Only two hernias were classified as femoral (2.2%). Symptoms such as local pain or discomfort were reported by 70 patients (77.7%).

**TABLE 1:** Demographic and clinical data.

Variable	All patients (N = 90)
	Mean (SD)
Age (years)	49.42 (±28.56)
BMI	25.9 (±3.5)
Gender	N (%)
Male	85 (94.4%)
Female	5 (5.6%)
Smoking	10 (11.1%)
Comorbidities	
No comorbidities	36 (40.0%)
Hypertension	29 (32.2%)
Diabetes Mellitus	7 (7.8%)
COPD	1 (1.1%)
Liver disease	1 (1.1%)
Kidney disease	1 (1.1%)
Aortic aneurysm	0 (0.0%)
Other comorbidities	27 (30.0%)
Non-inguinal chronic pain	
No pain	76 (84.4%)
Yes	16 (17.7%)
Hernia side	
Right hernia	34 (37.8%)
Left hernia	30 (33.3%)
Bilateral hernia	26 (28.9%)
Preoperative symptom	
Pain	5 (5,55%)
Discomfort Pain and disconfort	23 (25.55%) 42 (46.66%
Asymptomatic	20 (22.22%)
Preoperative Pain Numerical Scale	
Mild (1–3)	22 (24.44%)
Moderate (4–6)	28 (31.11%)
Severe (7–10)	19 (21.11%)
No Pain	21 (23.33%)

SD, standard deviation; BMI, body mass index; COPD, chronic obstructive pulmonary disease.

### Intraoperative and Post-Operative Outcomes

There were no intraoperative complications. During the first week after surgery, haematomas were observed in 17 patients (19.1%), all of which required no intervention and were classified as Clavien-Dindo I. Within the first month post-operatively, complications included seroma in 6 patients (6.7%), haematoma in 8 patients (9%), and intense pain in 3 patients (3.4%). These patients required only basic analgesia (e.g., dipyrone or paracetamol, and non-steroidal anti-inflammatory drugs) without the need for opioids or stronger pain medications. According to the Clavien-Dindo classification, 15 patients were classified as Grade I, 1 patient as Grade II, and 1 as Grade IIIb.

The mean duration of the surgery was 63.84 min (SD 21.19). Most patients were discharged within 12 h post-operatively (n = 80, 88%). The relative frequency of chronic pain from 3 months postoperatively was 3.52%. There were no hernia recurrences during the follow-up period. Intraoperative and post-operative data are presented in Table 2.

**TABLE 2:** Intraoperative data.

Variable	(N = 90 patients) N (%)
EHS Classification^a^ (N = 119 hernias)	
Medial	
M1	22 (24.44%)
M2	22 (24.44%)
M3	8 (8.89%)
Lateral	
L1	23 (25.56%)
L2	38 (42.44%)
L3	4 (4.44%)
Femoral	
F1	2 (2,22%)
Postoperative Pain Scale (7 days)	
Mild pain (1–3)	44 (51.15%)
Moderate pain (4–6)	15 (17.44%)
Severe pain (7–10) No Pain	6 (6.97%) 22 (25.58%)
Missing	4
Postoperative complications (7 days)	
No complications	66 (76.7%)
Bleeding	0 (0.0%)
Severe pain	0 (0.0%)
Hematoma	17 (19.8%)
Hematuria	0 (0.0%)
Others	4 (4.7%)
Clavien-Dindo I	17 (19.1%)
Missing	4
Postoperative complications (1 month)	
No complications	69 (80.23%)
Seroma	6 (7.0%)
Hematoma	8 (9.3%)
Severe pain	3 (3.5%)
Recurrence	0 (0.0%)
Clavien-Dindo I	15 (17.4%)
Clavien-Dindo II	1 (1,16%)
Clavien-Dindo IIIb	1 (1,16%)
Missing	4
Pain after 3 and 6 months (numeric scale 0–10)	
Pain at 3 months	3/85 (3.52%)
Pain at 6 months	4/81 (4.93%)
Hospital stay	
Day clinic	80/90 (89%)
1-night stay	10/90 (11%)

EHS, european hernia society.

^a^ Classified according to the European Hernia Classification.

The missing cases, which did not respond to follow-up, were excluded from the statistical analysis.

### EuraHS-QoL Quality of Life Before and After Surgery

The mean pre-operative EuraHS-QoL score was 19.79 (SD 15.84), with a median of 17.33 (IQR 6.75–30.99), a minimum of 0, and a maximum of 65. One month post-operatively, the mean score was 18.76 (SD 13.66), median 16.25 (IQR 9.00–26.91), with scores ranging from 0 to 62. At 3 months, the mean score was 4.5 (SD 9.12), median 1.00 (IQR 0–5.00), range 0–67.33. At 6 months, the mean score was 3.93 (SD 8.07), median 0 (IQR 0–5.50), range 0–48.

**FIGURE 2:**
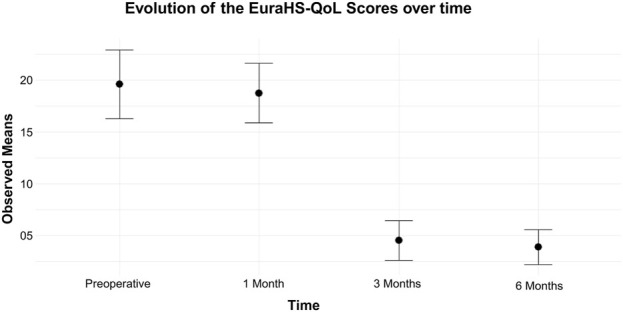
Evolution of the EuraHS-QoL score over time.

Analysis of the EuraHS-QoL score over time across the four time points (pre-operative, 1 month, 3 months, and 6 months) showed no significant difference between the pre-operative score and the score at 1 month post-operatively. However, the differences between the pre-operative score and those at 3 and 6 months post-operatively were statistically significant (p < 0.001), indicating a significant improvement in quality of life from the third month onward Figure 2.

## Discussion

The assessment of quality of life after laparoscopic inguinal hernia repair has gained importance, extending beyond the simple presence or absence of recurrence and procedural complications. The development of hernia-specific quality of life questionnaires has proven to be more effective in evaluating patients pre- and post-operatively than generic instruments such as the SF-36. Disease-specific tools, like the Carolina Comfort Score and EuraHS-QoL [16, 28], used as patient-reported outcome measures (PROMs), represent a paradigm shift—especially for oligosymptomatic or asymptomatic patients.

Filip et al., in their European validation study of the EuraHS-QoL instrument, demonstrated it to be a valid, reliable tool for measuring quality of life in patients undergoing laparoscopic inguinal hernia repair. It is easy to apply both pre- and post-operatively. They observed a significant improvement in quality of life from 3 weeks post-surgery, which continued to improve up to 3 months and then stabilized with a slight decline in scores up to 12 months [16].

The Portuguese Collaborative Research Group, in a prospective multicentre study evaluating EuraHS-QoL in 893 patients undergoing open Liechtenstein inguinal hernia repair, also observed a significant improvement in quality of life up to 3 months post-operatively. Additionally, they identified that poor pre-operative quality of life, non-absorbable mesh fixation, immediate post-operative pain, minor complications, and younger age were associated with lower quality of life at 3 months.

Konrad et al., in a prospective randomised clinical trial comparing non-fixation techniques (Progrip Lap and lightweight mesh without fixation) versus fixation using staples for laparoscopic TEP hernia repair, found no significant differences between groups in terms of acute or chronic pain, recurrence, length of hospital stay, or time to return to normal activities. All groups showed significant improvement in EuraHS-QoL scores after 12 months [32, 33].

Sanderson et al. compared EuraHS-QoL outcomes between laparoscopic and open (Lichtenstein) hernia repair techniques and found no significant differences between surgical approaches in terms of pain, restriction, or cosmetic domains [26].

Shukla et al. defined the minimum clinically important difference (MCID) for the EuraHS-QoL questionnaire as follows: 3 points for the pain domain, 5 points for the restriction domain, 2 points for the cosmetic domain, and 10 points for the total EuraHS-QoL score [34].

Our study demonstrated a significant improvement in EuraHS-QoL scores beginning at 3 months, remaining stable through 6 months of follow-up. All three domains improved significantly over time, corroborating previous results that validated the instrument’s sensitivity and clinical utility in monitoring quality of life post-hernia repair.

In our study, the total EuraHS-QoL score showed significant improvement across all three domains during follow-up, corroborating previous findings validating the sensitivity and reliability of the EuraHS-QoL score in multiple clinical contexts. At the 1-month mark, however, no significant improvement in the total score was observed compared to the pre-operative baseline. This may be attributable to the restriction domain, as patients are advised to avoid sports activities for 30 days and may self-limit more intense physical efforts. In our study, the pain and cosmetic domains showed significant improvement within the first month, while the restriction domain score increased by 2.94 points relative to the pre-operative period, differing from findings in the literature. Our study also confirmed previously observed associations between higher pre-operative and immediate post-operative pain levels and poorer quality-of-life scores using the EuraHS-QoL tool.

Chronic post-operative pain remains a clinically relevant condition even in laparoscopic surgeries. Reinpold et al., in a systematic review, reported chronic pain rates of 18% for open inguinal hernia repair (ranging from 0.7% to 75%) and 6% for laparoscopic repair (ranging from 1% to 16%). Well-established risk factors included female sex, younger age, high pre-operative pain intensity, immediate post-operative pain, and recurrence surgeries [23]. Chu et al., in another systematic review and meta-analysis, found an overall chronic pain incidence of 17.1%, regardless of technique. Their article points out the lack of consensus on the definition of chronic pain—while the International Association for the Study of Pain (IASP) defines it as pain lasting more than 3 months, some authors suggest extending the threshold to 6 months due to the prolonged inflammatory response caused by mesh implants [35]. Alabi et al. and Techapongsatorn et al., in umbrella reviews, found reduced chronic pain with the use of glue compared to tacks. Wang et al., in a meta-analysis, observed lower chronic pain rates with self-fixating meshes versus conventional meshes, although they noted the heterogeneity of surgical techniques and mesh fixation methods as a limitation [13, 17, 24]. In our study, the chronic pain rate was among the lowest reported in the literature.

Among the limitations of this study are the short follow-up period, which prevents the assessment of hernia recurrence and long-term pain, and the small sample size, which restricts the ability to extrapolate analyses of risk factors for chronic pain.

### Conclusion

Trans-abdominal preperitoneal laparoscopic inguinal hernia repair using self-fixating mesh had a positive impact on the quality-of-life improvement during the 6-month post-operative follow-up.

## Data Availability

The raw data supporting the conclusions of this article will be made available by the authors, without undue reservation.

## Footnotes

1
https://www.enterprise-development.org/measuring-results-the-dced-standard/sample-size-calculator/
